# Acneiform rash secondary to trametinib in two patients with neurofibromatosis^☆^

**DOI:** 10.1016/j.abd.2025.501158

**Published:** 2025-07-03

**Authors:** Macarena Nougues, Luciana Laura Tirelli, Paula Carolina Luna, Darío Macas Ordoñez, Margarita Larralde

**Affiliations:** Dermatology Departament, Hospital Aleman de Buenos Aires, Ciudad Autónoma de Buenos Aires, CABA, Argentina

Dear Editor,

Trametinib is a reversible, selective inhibitor of Mitogen-activated protein Kinase (MEK) blocking enzyme phosphorylation and activation, affecting both the proliferation and survival of tumor cells.^1^

The most commonly reported side effects of trametinib are dermatologic; acneiform eruptions are the most prevalent. In these cases, the dose of the MEK inhibitor can be reduced; nevertheless, this may be associated with decreased treatment efficacy.

We report two cases of acneiform rash secondary to trametinib.

A 14-year-old male diagnosed with NF1 at age 4, presented an optic chiasm glioma, associated with two extensive, plexiform neurofibromas (PNF). One neurofibroma was located on the right foot, causing an inoperable deformity. The other, emerged from the second branch of the right trigeminal nerve (V2), producing facial disfigurement. It was also inoperable. Medical treatment with trametinib was started. After a year of treatment, the facial mass had decreased by 30% in size. Upon reaching the maximum dose of trametinib, the patient developed an acneiform eruption (Fig. 1), affecting the nasal region, forehead, upper eyelid, cheeks, and chin. He received topical treatment with clindamycin and benzoyl peroxide, in addition to reducing the dose of trametinib. As papules and pustules persisted, oral treatment with doxycycline was initiated. After evidencing clinical improvement, the trametinib dose was increased without presenting further complications. To date, oncological treatment is ongoing, and a mild dermatitis persists, evidencing no other adverse events.

**Figure 1:**
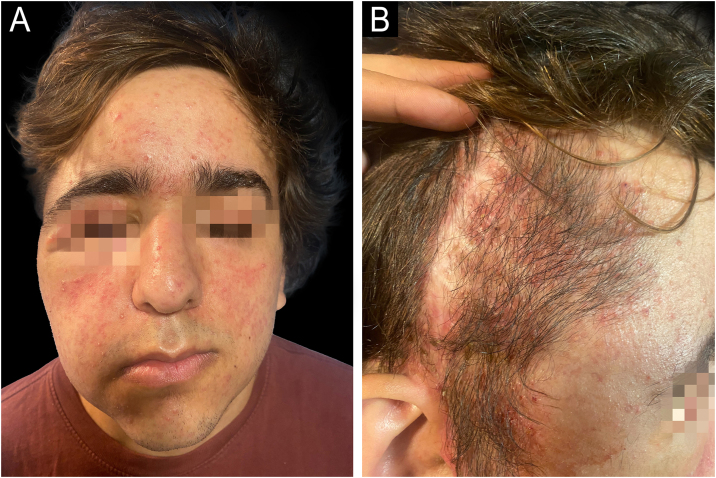
In the left picture it can be seen Patient 1 presenting generalized comedones, papules and pustules all over his face. In the right picture, the acneiform rash also compromises the right parietotemporal scalp.

The second patient is a 21-year-old female diagnosed with NF1 during her first year of life, presenting a left orbital neurofibroma. The tumor was resected on five occasions; unfortunately, it recurred. Onco-specific treatment with trametinib was then initiated. A month later, she presented an acneiform rash, involving her face and back (Fig. 2). She started treatment with lymecycline, as well as a reduction of trametinib. This treatment was successful, although when tetracyclines were discontinued the papulopustular eruption reappeared. Lymecycline was reintroduced alongside a progressive increase in the dose of trametinib. This treatment was well tolerated and resulted in both aesthetic and clinical improvement

**Figure 2:**
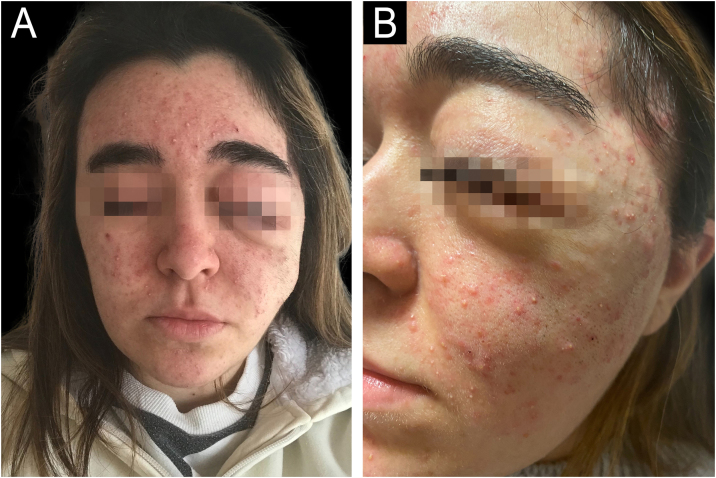
In the left picture, on a frontal view, it can be seen Patient 2 presenting an acneiform rash on her face. In the right picture, a close-up, it can be seen in detail multiple papules and pustules just like Patient 1.

Two years later, trametinib was discontinued as the lesion remained stable and Lymecycline was also suspended without relapse.

Trametinib is a selective, reversible inhibitor of MEK1/2 activation and kinase activity.^2^ Several studies have shown the efficacy of MEK inhibitors in patients with NF-1 associated with non-surgical low-grade gliomas and PNF.

NF1 is caused by the loss of neurofibromin, the protein product of the *NF1* gene. Neurofibromin is a ras-GAP protein, a negative regulator of RAS signaling. Loss of functional neurofibromin results in activation of the classic RAS-MAPK signaling cascade, cell proliferation, and subsequent tumor formation.

MEK inhibitors have been found to stabilize or even reduce the volume of these tumors. During the course of therapy, many patients display side effects.^3^ Acneiform eruptions are the most common dermatologic side effect, occurring in 77% of the population studied by Anforth et al.^1^ It is a dose-dependent follicular, papulopustular eruption compromising the face, scalp, chest, and upper back.^4^

The precise mechanism which triggers this side effect is not clear. What is known is that insulin-like growth factor-1 induces sebaceous gland lipogenesis via sterol response element-binding protein-1, which is activated by the Phosphatidylinositol 3-Kinase (PI3K)/protein Kinase B (AKT) signaling pathway, and is related to the pathogenesis of acne. Unknown interactions between BRAF-MEK-ERK and PI3K-AKT pathways might also be responsible for this effect.^1^

The lesions usually develop in the initial 2 weeks of treatment, worsening during the first month. Mild cases are treated with twice-daily dosing of topical antibiotics. For moderate cases, systemic tetracyclines are recommended. Topical steroids can be added if a severe inflammatory reaction is present. Refractory patients could be treated orally with antibiotics and steroids.

Based on the response to the established treatment, the reduction of the oncology drug dose should also be considered. Severe refractory cases might even require suspending oncologic treatment.^5^, ^6^ Effective treatment may be continued for the duration of the trametinib therapy.^1^ It is important to highlight that these reactions are associated with good antitumor activity.

To conclude, MEK inhibitors have shown good results in the treatment of neurologic-associated tumors. Effective treatment should aim to ideally not decrease the dose of trametinib, but should be considered in refractory cases, requiring a mandatory multidisciplinary approach between oncology and dermatology specialists.

## Financial support

None declared.

## Authors' contributions

Macarena Nougues: Manuscript writing and editing; data analysis.

Luciana Laura Tirelli: Manuscript writing and editing; supervision of the project.

Paula Carolina Luna: Data collection; revision of the manuscript.

Darío Macas Ordoñez: Manuscript editing; data visualization and graphical layout.

Margarita Larralde: Critical review and revision of the manuscript.

All authors reviewed and approved the final version of the manuscript.

## Conflicts of interest

None declared.

## References

[1] Anforth R., Liu M., Nguyen B., Uribe P., Kefford R., Clements A. (2014). Acneiform eruptions: a common cutaneous toxicity of the MEK inhibitor trametinib. Australas J Dermatol.

[2] Ronsley R., Hounjet C.D., Cheng S., Rassekh S.R., Duncan W.J., Dunham C. (2021). Trametinib therapy for children with neurofibromatosis type 1 and life-threatening plexiform neurofibroma or treatment-refractory low-grade glioma. Cancer Med.

[3] Manousaridis I., Mavridou S., Goerdt S., Leverkus M., Utikal J. (2013). Cutaneous side effects of inhibitors of the RAS/RAF/MEK/ERK signalling pathway and their management. J Eur Acad Dermatol Venereol.

[4] Fabbrocini G., Panariello L., Caro G., Cacciapuoti S. (2015). Acneiform rash induced by EGFR inhibitors: review of the literature and new insights. Skin Appendage Disord.

[5] Andrews E.D., Garg N., Patel A.B. (2020). A retrospective chart review on oral retinoids as a treatment for epidermal growth factor receptor inhibitor and mitogen-activated protein kinase kinase inhibitor induced acneiform eruptions. J Am Acad Dermatol.

[6] Reyes-Habito C.M., Roh E.K. (2014). Cutaneous reactions to chemotherapeutic drugs and targeted therapies for cancer. Part II. Targeted therapies. J Am Acad Dermatol.

